# Supplementary material for: Fertilization controls tiller numbers via transcriptional regulation of a MAX1-like gene in rice cultivation
Source: Nat Commun. 2023 Jun 8;14:3191. doi: 10.1038/s41467-023-38670-8 (PMC10250342; doi:10.1038/s41467-023-38670-8)
Supplement: Supplementary file 1 — Supplementary Information [file 41467_2023_38670_MOESM1_ESM.pdf]

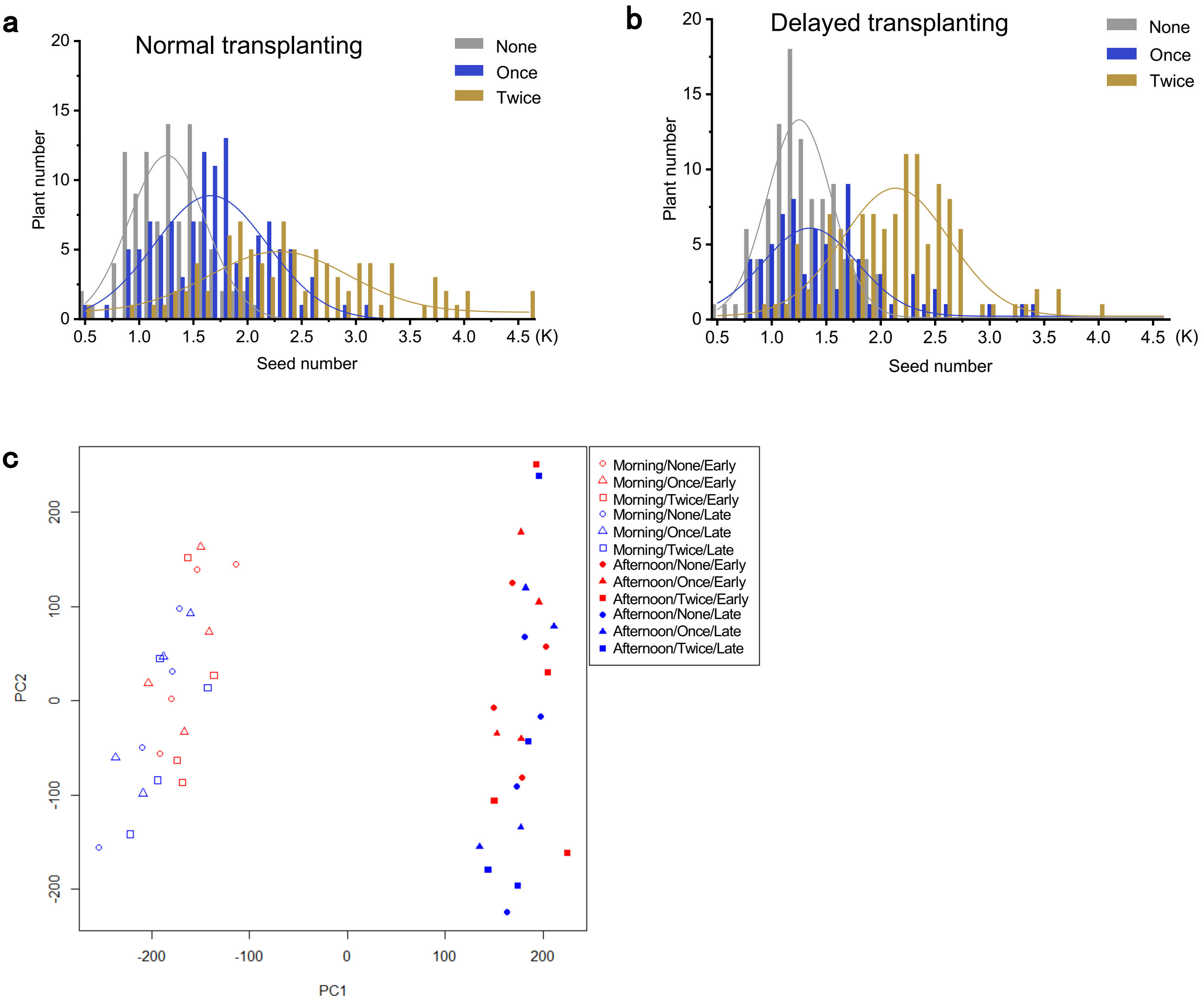

Supplementary Fig.1 Field transcriptome data. Total seed number of per plant under different fertilization condition (a. normal transplanting, b. delayed transplanting). c. Principal component analysis of gene expression, corresponding to Supplementary Data 1.

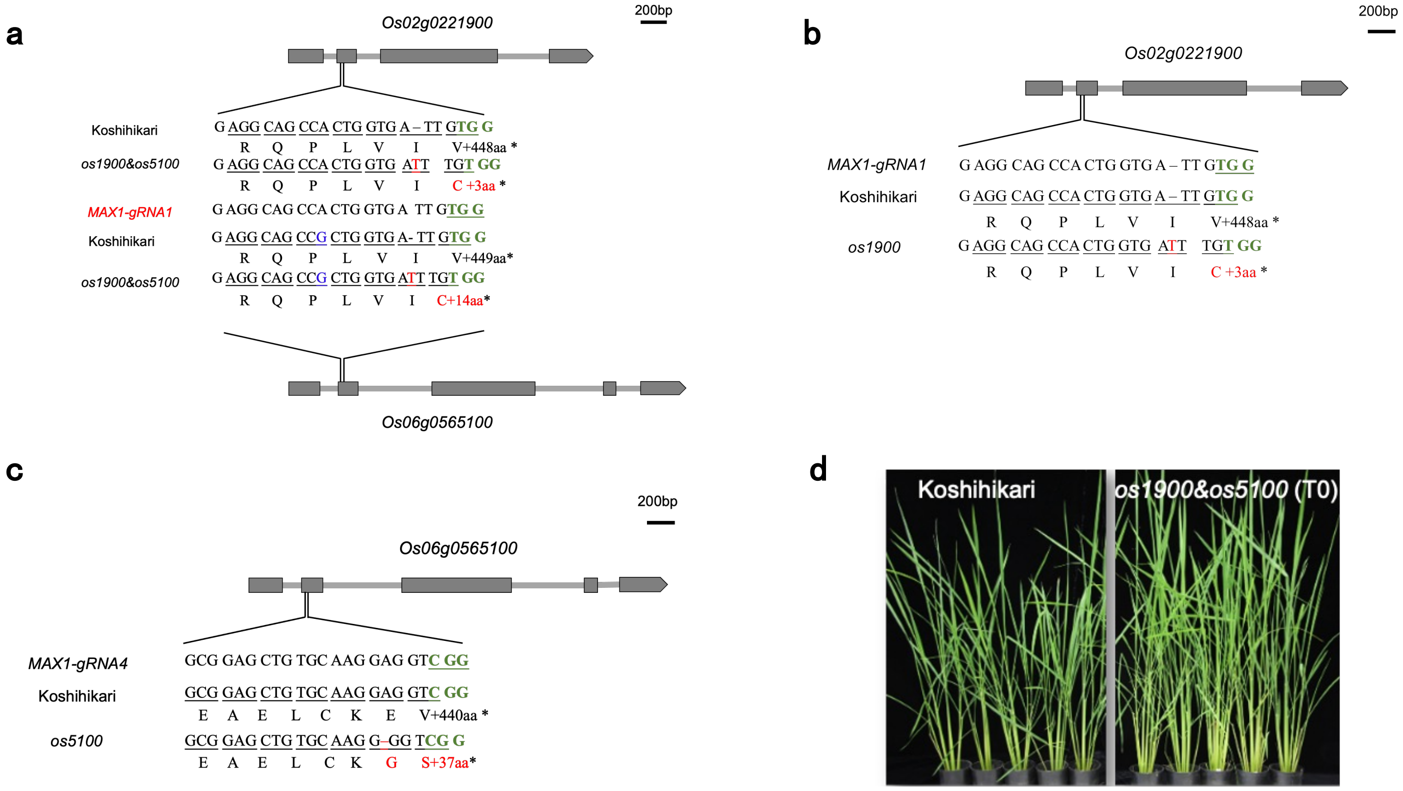

Supplementary Fig. 2 Amino acid sequences of various kinds of *MAX1*-like mutants. *os1900 & os5100* double mutant (a), *os1900* single mutant (b), *os5100* single mutant (c), *os1900&os5100* phenotype of T0 generation (d).

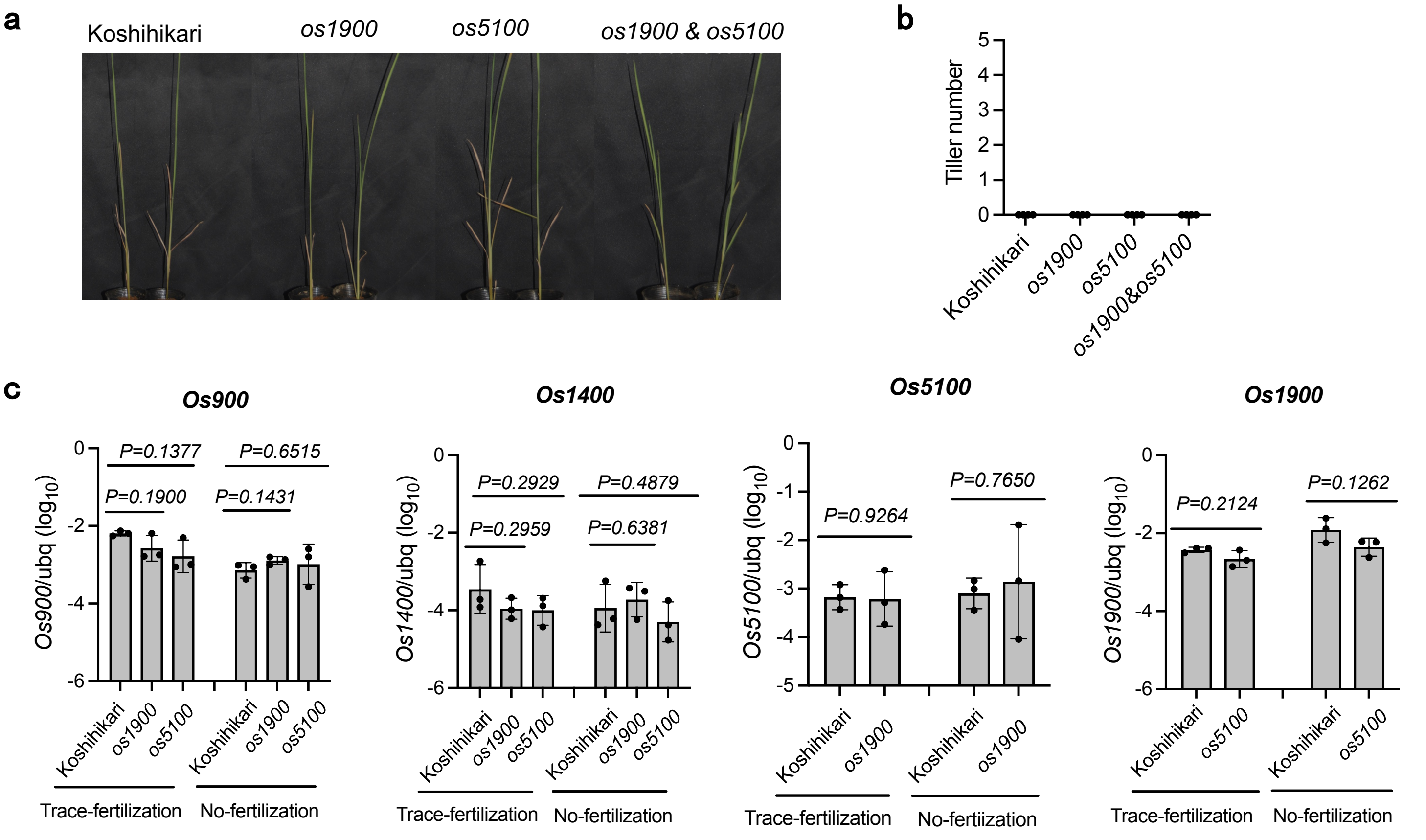

Supplementary Fig. 3 Expression of *MAX1*-like genes in *os1900*, *os5100* single mutants under trace and no fertilizer conditions**.** (a, b) Phenotype of *os1900*, *os5100* single mutants under no fertilization condition. n=5. (c) *MAX1*-like genes expression in *os1900*, *os5100* single mutants in shoot, Error bar: SD, n=3 biologically independent samples, significance values are from Student's *t*-test (two-tailed).

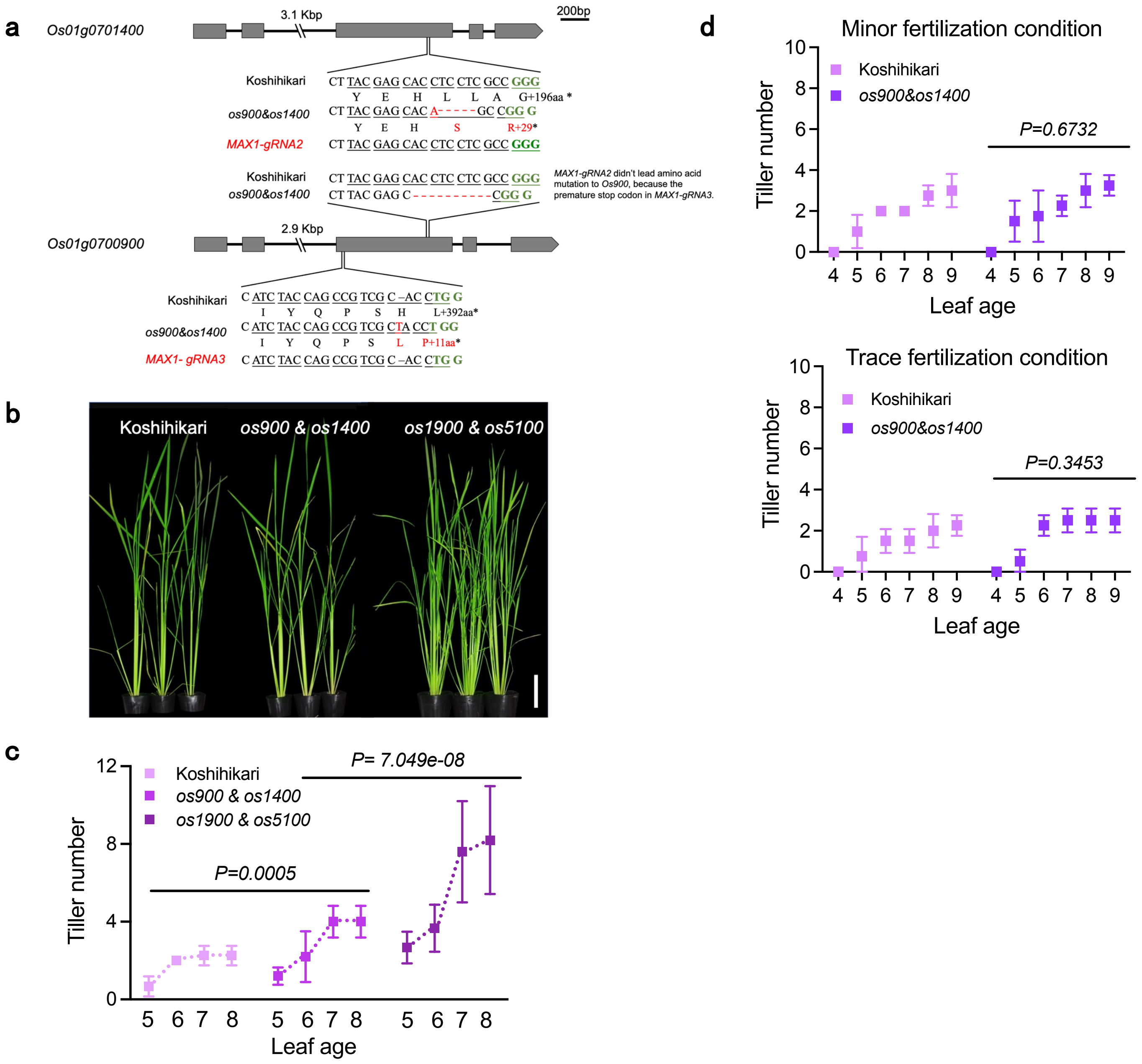

Supplementary Fig. 4 *os900&os1400* gRNA target sequence sketch (a) and their phenotype (b,c) under normal condition. Tiller number in *os900&os1400* under minor-/trace- fertilization conditions (d). n=5,6 biologically independent plants (c). n=4 biologically independent plants (d). P-values of different systems were obtained from generating a GLM model and subsequent ANOVA test. Error bar: SD.

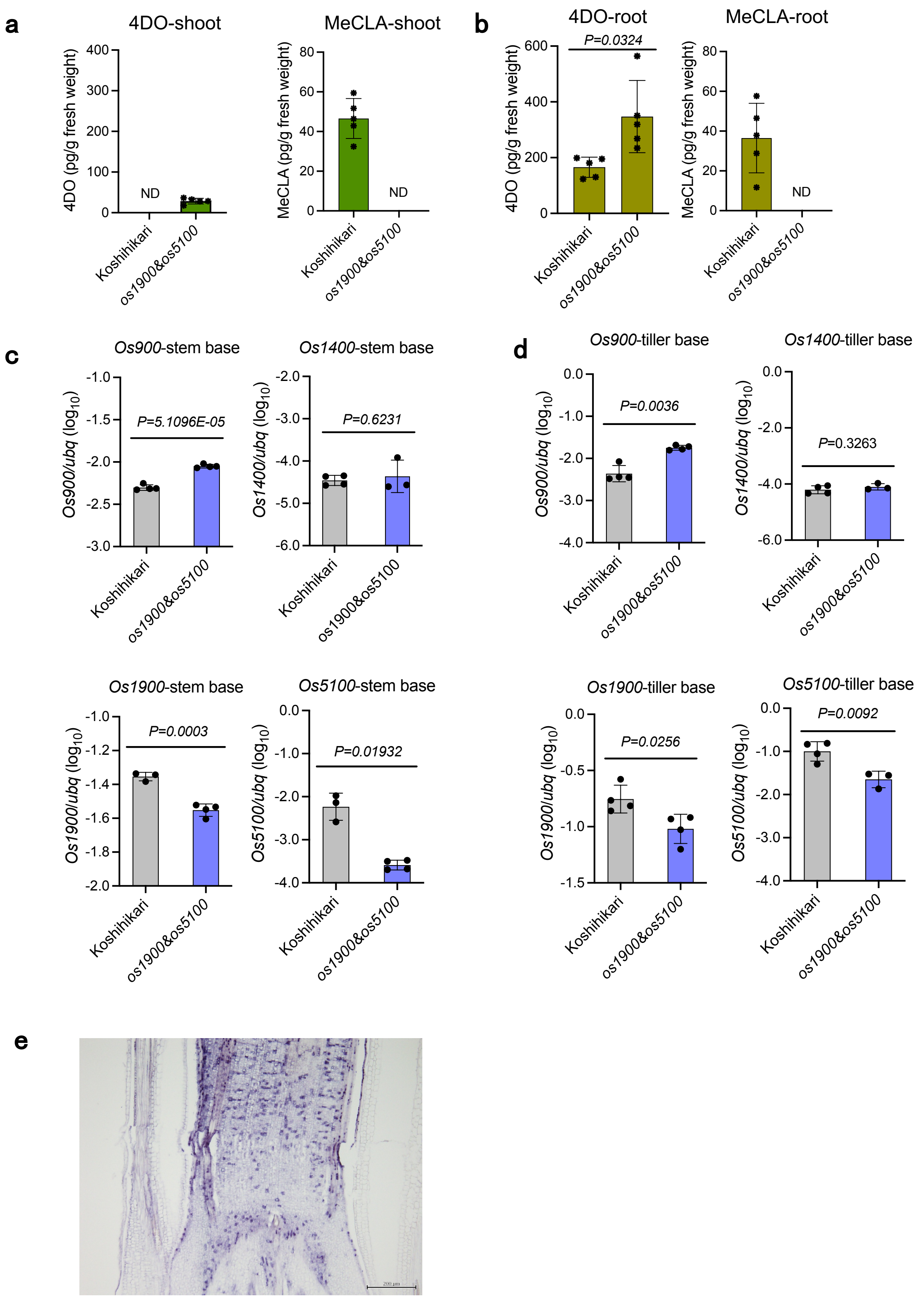

Supplementary Fig. 5 Endogenous levels of SLs (pg/gFW) and *MAX1*-like expression in shoot and root of Koshihikari and *os1900 & os5100* mutants. Result of 4DO, MeCLA in shoot (a) and root (b). ND: not detected, n = 5 biologically independent samples. (c) *MAX1*-like genes expression in stem base of Koshihikari and *os1900&os5100* mutants. (d) *MAX1*-like genes expression of the first tiller base of Koshihikari and *os1900&os5100* mutants, the samples were the same with that for RNA-seq. n = 3,4 biologically independent samples, (c,d). (a-d) P-values from Student's t-test, at 0.05 level, Error bar is SD, significance values are from Student's *t*-test (two-tailed). (e) Expression of histone H4 (positive control) *in situ* analysis.

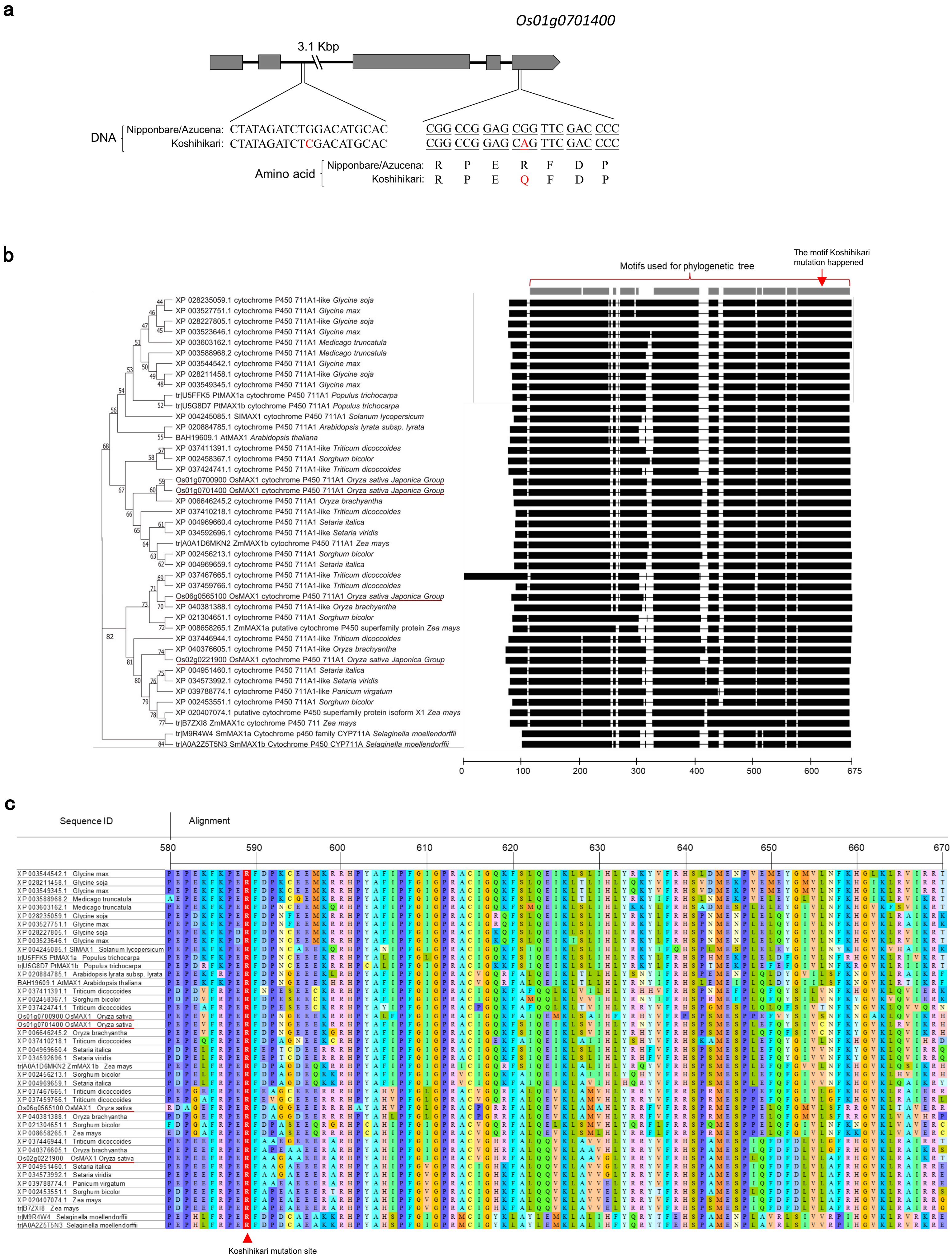
a
Os01g0701400
3.1 Kbp
DNA
Nipponbare/Azucena: CTATAGATCTGGACATGCAC
Koshihikari: CTATAGATCTCGACATGCAC
CGG CCG GAG CGG TTC GAC CCC
CGG CCG GAG CAG TTC GAC CCC
Amino acid
Nipponbare/Azucena: R P E R F D P
Koshihikari: R P E Q F D P
b
Motifs used for phylogenetic tree
The motif Koshihikari mutation happened
XP 028235059.1 cytochrome P450 711A1-like Glycine soja
XP 003527751.1 cytochrome P450 711A1 Glycine max
XP 028227805.1 cytochrome P450 711A1-like Glycine soja
XP 003523646.1 cytochrome P450 711A1 Glycine max
XP 003603162.1 cytochrome P450 711A1 Medicago truncatula
XP 003588968.2 cytochrome P450 711A1 Medicago truncatula
XP 003544542.1 cytochrome P450 711A1 Glycine max
XP 028211458.1 cytochrome P450 711A1-like Glycine soja
XP 003549345.1 cytochrome P450 711A1 Glycine max
tr|U5FFK5 PtMAX1a cytochrome P450 711A1 Populus trichocarpa
tr|U5G8D7 PtMAX1b cytochrome P450 711A1 Populus trichocarpa
XP 004245085.1 SlMAX1 cytochrome P450 711A1 Solanum lycopersicum
XP 020884785.1 cytochrome P450 711A1 Arabidopsis lyrata subsp. lyrata
BAH19609.1 AtMAX1 Arabidopsis thaliana
XP 037411391.1 cytochrome P450 711A1-like Triticum dicoccoides
XP 002458367.1 cytochrome P450 711A1 Sorghum bicolor
XP 037424741.1 cytochrome P450 711A1-like Triticum dicoccoides
Os01g0700900 OsMAX1 cytochrome P450 711A1 Oryza sativa Japonica Group
Os01g0701400 OsMAX1 cytochrome P450 711A1 Oryza sativa Japonica Group
XP 006646245.2 cytochrome P450 711A1 Oryza brachyantha
XP 037410218.1 cytochrome P450 711A1-like Triticum dicoccoides
XP 004969660.4 cytochrome P450 711A1 Setaria italica
XP 034592696.1 cytochrome P450 711A1-like Setaria viridis
tr|A0A1D6MKN2 ZmMAX1b cytochrome P450 711A1 Zea mays
XP 002456213.1 cytochrome P450 711A1 Sorghum bicolor
XP 004969659.1 cytochrome P450 711A1 Setaria italica
XP 037467665.1 cytochrome P450 711A1-like Triticum dicoccoides
XP 037459766.1 cytochrome P450 711A1-like Triticum dicoccoides
Os06g0565100 OsMAX1 cytochrome P450 711A1 Oryza sativa Japonica Group
XP 040381388.1 cytochrome P450 711A1-like Oryza brachyantha
XP 021304651.1 cytochrome P450 711A1 Sorghum bicolor
XP 008658265.1 ZmMAX1a putative cytochrome P450 superfamily protein Zea mays
XP 037446944.1 cytochrome P450 711A1-like Triticum dicoccoides
XP 040376605.1 cytochrome P450 711A1-like Oryza brachyantha
Os02g0221900 OsMAX1 cytochrome P450 711A1 Oryza sativa Japonica Group
XP 004951460.1 cytochrome P450 711A1 Setaria italica
XP 034573992.1 cytochrome P450 711A1-like Setaria viridis
XP 039788774.1 cytochrome P450 711A1-like Panicum virgatum
XP 002453551.1 cytochrome P450 711A1 Sorghum bicolor
XP 020407074.1 putative cytochrome P450 superfamily protein isoform X1 Zea mays
tr|B7ZXI8 ZmMAX1c cytochrome P450 711 Zea mays
tr|M9R4W4 SmMAX1a Cytochrome p450 family CYP711A Selaginella moellendorffii
tr|A0A2Z5T5N3 SmMAX1b Cytochrome P450 CYP711A Selaginella moellendorffii
0 100 200 300 400 500 600 675
c
Sequence ID
Alignment
580 590 600 610 620 630 640 650 660 670
Koshihikari mutation site

d

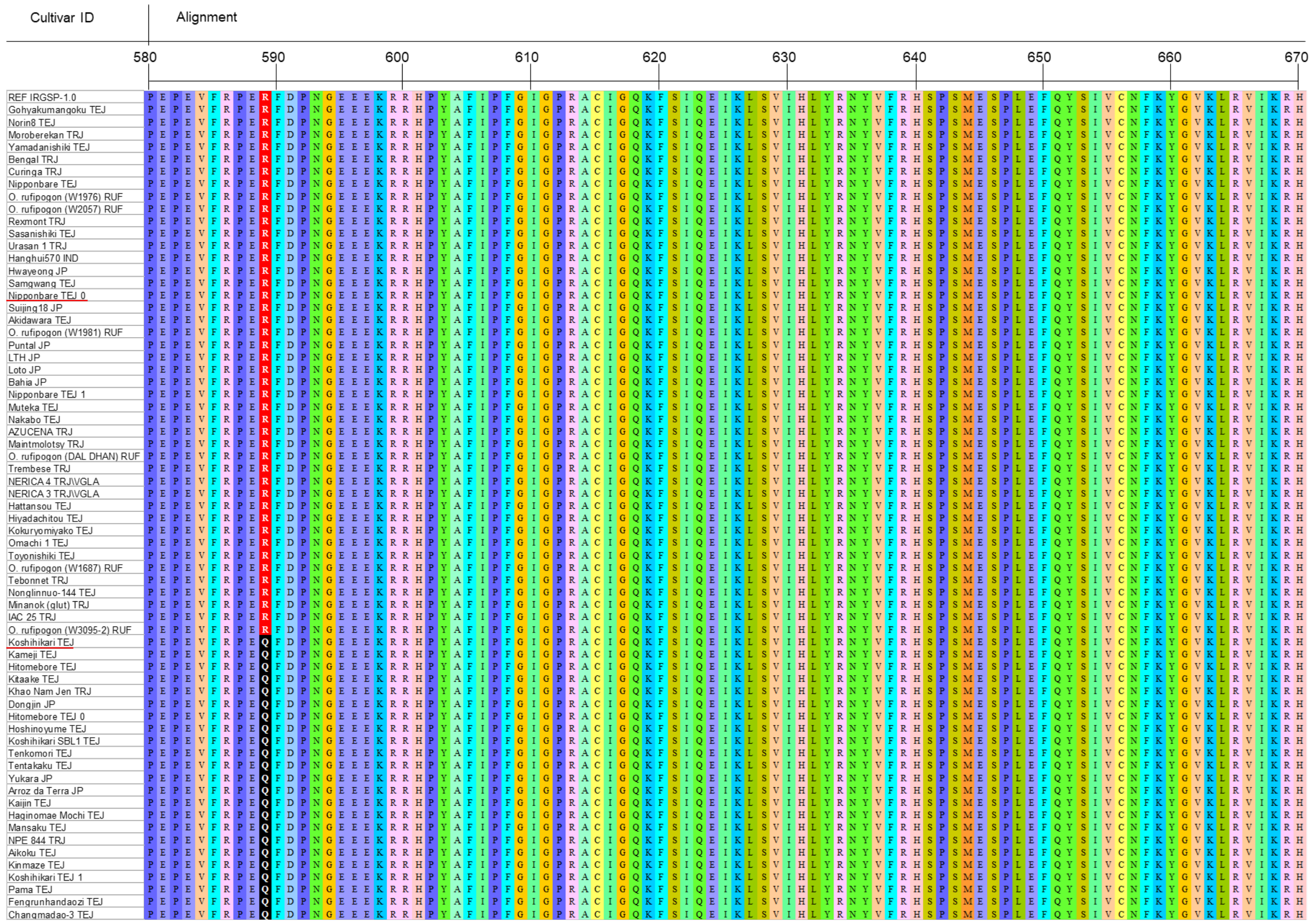

Supplementary Fig. 6 Conservation analysis of amino acid mutation sites in *MAX1* homologs, which happened in Koshihikari.

a. Sequence alignment of *Os1400* in Nipponbare, Azucena and Koshihikari.

b. Phylogenetic tree and amino acid conservation analysis of *MAX1* homologs. The amino acid sequences of the *MAX1*-like genes are from NCBI and Uniprot. Phylogenetic tree is by neighbor-joining method.

c. Conservative property exhibition of motif which the mutation site happened in Koshihikari.

d. Amino acid mutation of Koshihikari analysis in various types rice. Sequence data was from TASUKE database.

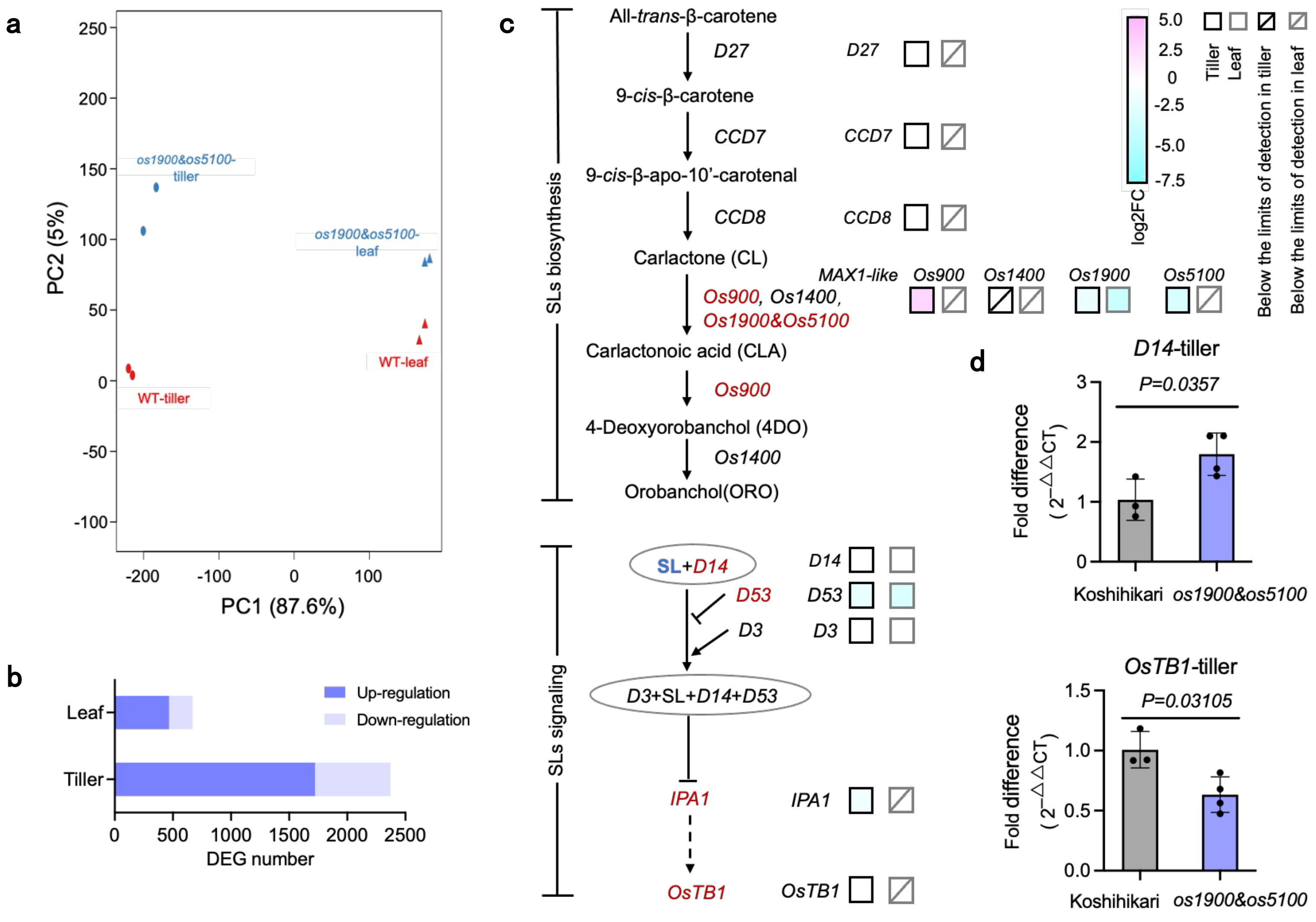

Supplementary Fig. 7 Differential gene expression in the first tiller base and leaf of Koshihikari and *os1900&os5100* mutant in SL metabolic and signal transduction. Global expression data was obtained by RNA-seq, FDR<0.05. (a) The PCA analysis of all genes in RNA-seq result. (b) Number of differentially expressed genes (DEGs) in tiller base and leaf of RNA-seq. (c) Schematic diagram of SL biosynthesis and SL signaling pathways. Genes marked in red are those up-/down-regulated ones by >2-fold change in *os1900&os5100* mutants. (d) Expression of *D14* and *OsTB1* mRNA in the first tiller base of WT and *os1900&os5100* mutant by real-time PCR. P-values from Student's *t*-test. Error bars: SD. n = 3,4 biologically independent samples.

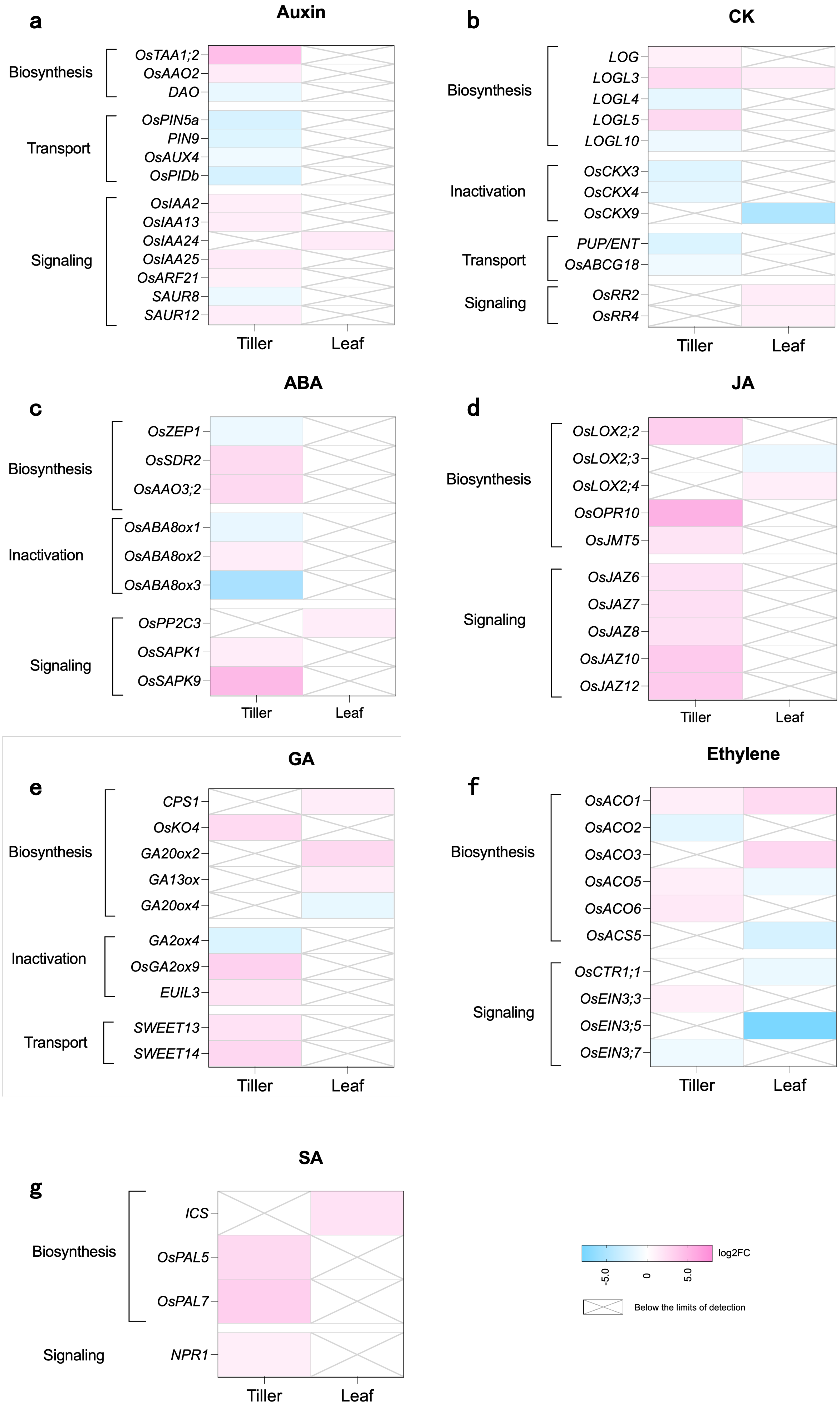
a
Auxin
Biosynthesis
OsTAA1;2
OsAAO2
DAO
Transport
OsPIN5a
PIN9
OsAUX4
OsPIDb
Signaling
OsIAA2
OsIAA13
OsIAA24
OsIAA25
OsARF21
SAUR8
SAUR12
Tiller
Leaf
b
CK
Biosynthesis
LOG
LOGL3
LOGL4
LOGL5
LOGL10
Inactivation
OsCKX3
OsCKX4
OsCKX9
Transport
PUP/ENT
OsABCG18
Signaling
OsRR2
OsRR4
Tiller
Leaf
c
ABA
Biosynthesis
OsZEP1
OsSDR2
OsAAO3;2
Inactivation
OsABA8ox1
OsABA8ox2
OsABA8ox3
Signaling
OsPP2C3
OsSAPK1
OsSAPK9
Tiller
Leaf
d
JA
Biosynthesis
OsLOX2;2
OsLOX2;3
OsLOX2;4
OsOPR10
OsJMT5
Signaling
OsJAZ6
OsJAZ7
OsJAZ8
OsJAZ10
OsJAZ12
Tiller
Leaf
e
GA
Biosynthesis
CPS1
OsKO4
GA20ox2
GA13ox
GA20ox4
Inactivation
GA2ox4
OsGA2ox9
EUIL3
Transport
SWEET13
SWEET14
Tiller
Leaf
f
Ethylene
Biosynthesis
OsACO1
OsACO2
OsACO3
OsACO5
OsACO6
OsACS5
Signaling
OsCTR1;1
OsEIN3;3
OsEIN3;5
OsEIN3;7
Tiller
Leaf
g
SA
Biosynthesis
ICS
OsPAL5
OsPAL7
Signaling
NPR1
Tiller
Leaf
log2FC
-5.0
0
5.0
Below the limits of detection

Supplementary Fig. 8 Differentially expressed genes of biosynthetic and signaling pathways related to phytohormones such as (a) Auxin, (b) CK, (c) ABA, (d) JA, (e) GA, (f) Ethylene, and (g) SA between WT and *os1900&os5100* mutants. RNA-seq data. FDR<0.05 in QLFtest. Only expressing genes in RNA-seq data are presented here. DEGs related to BR were not identified.

.

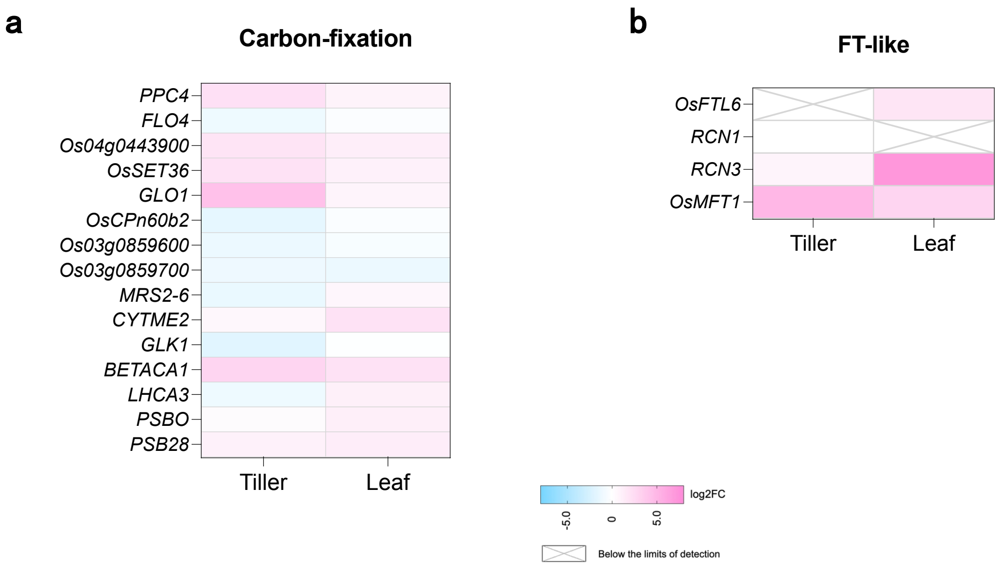

Supplementary Fig. 9 Differentially expressed genes of carbon-fixation related genes (a) and *FT*-like genes (b) in the first tiller base and leaf in *os1900&os5100* mutants compared with WT. Only expressing genes in RNA-seq data are presented here. QLFtest FDR<0.05.

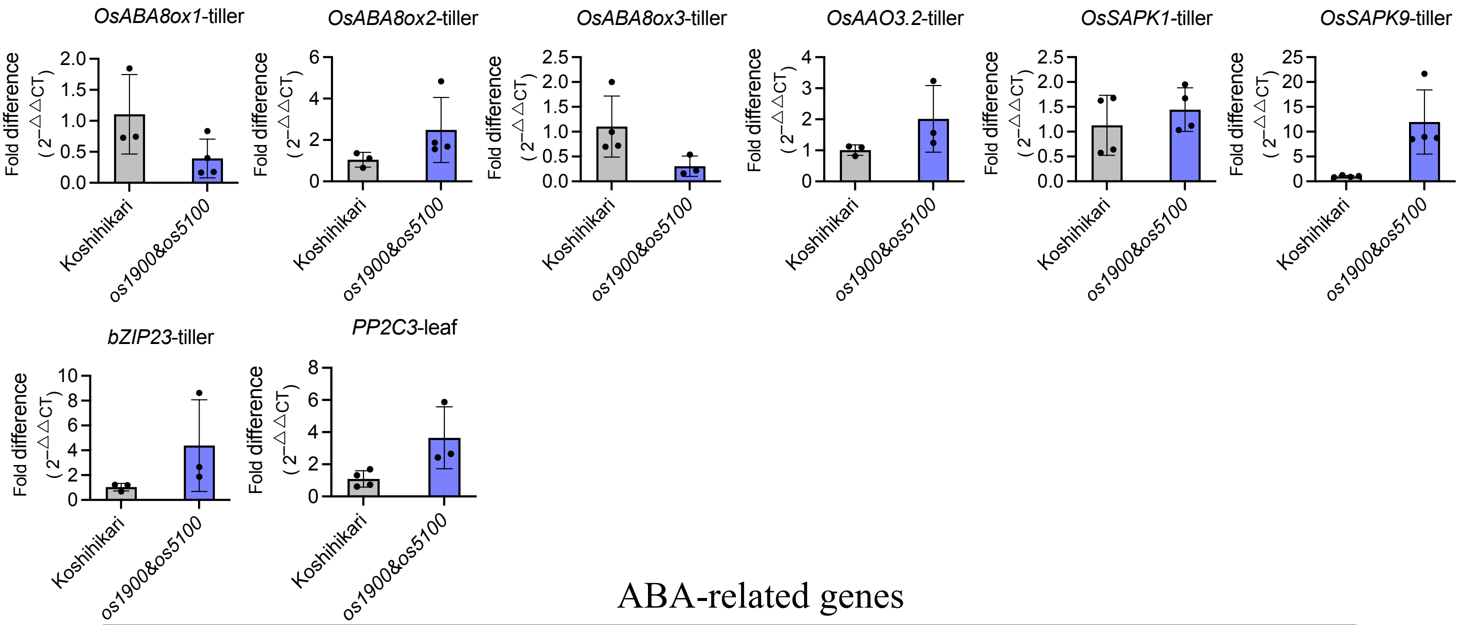

ABA-related genes

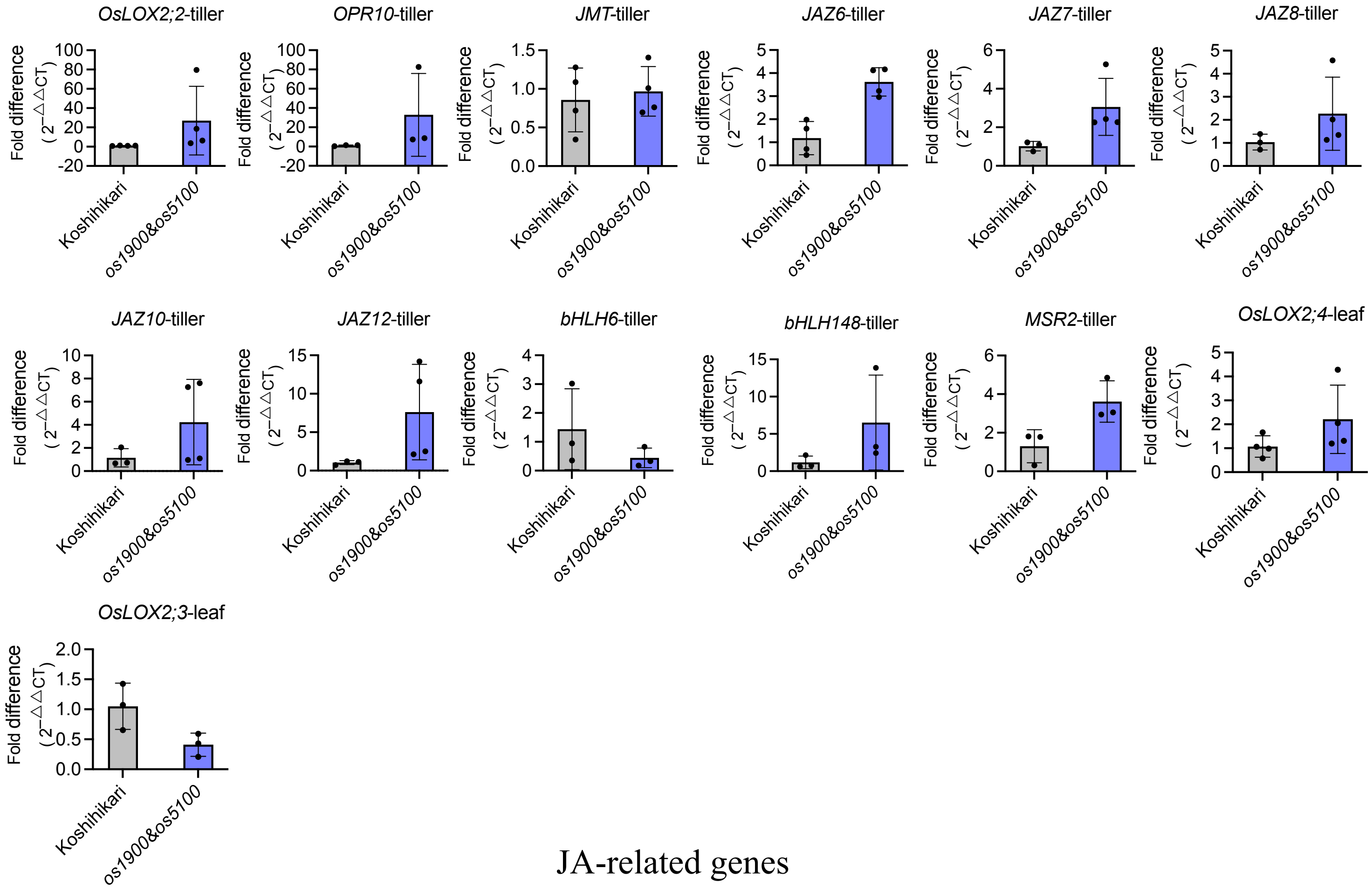

JA-related genes

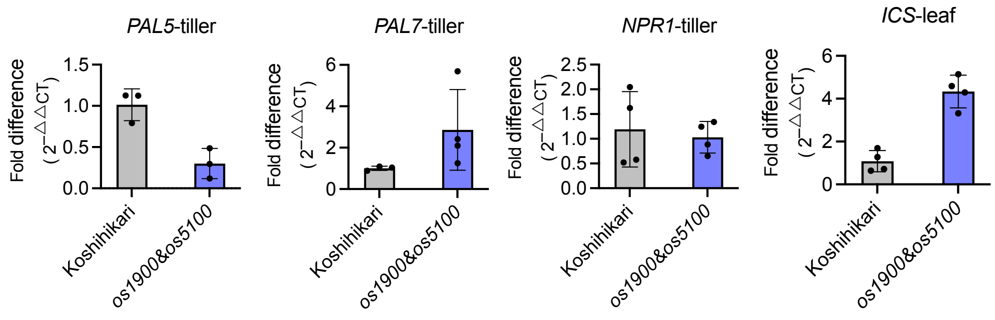

SA-related genes

continued

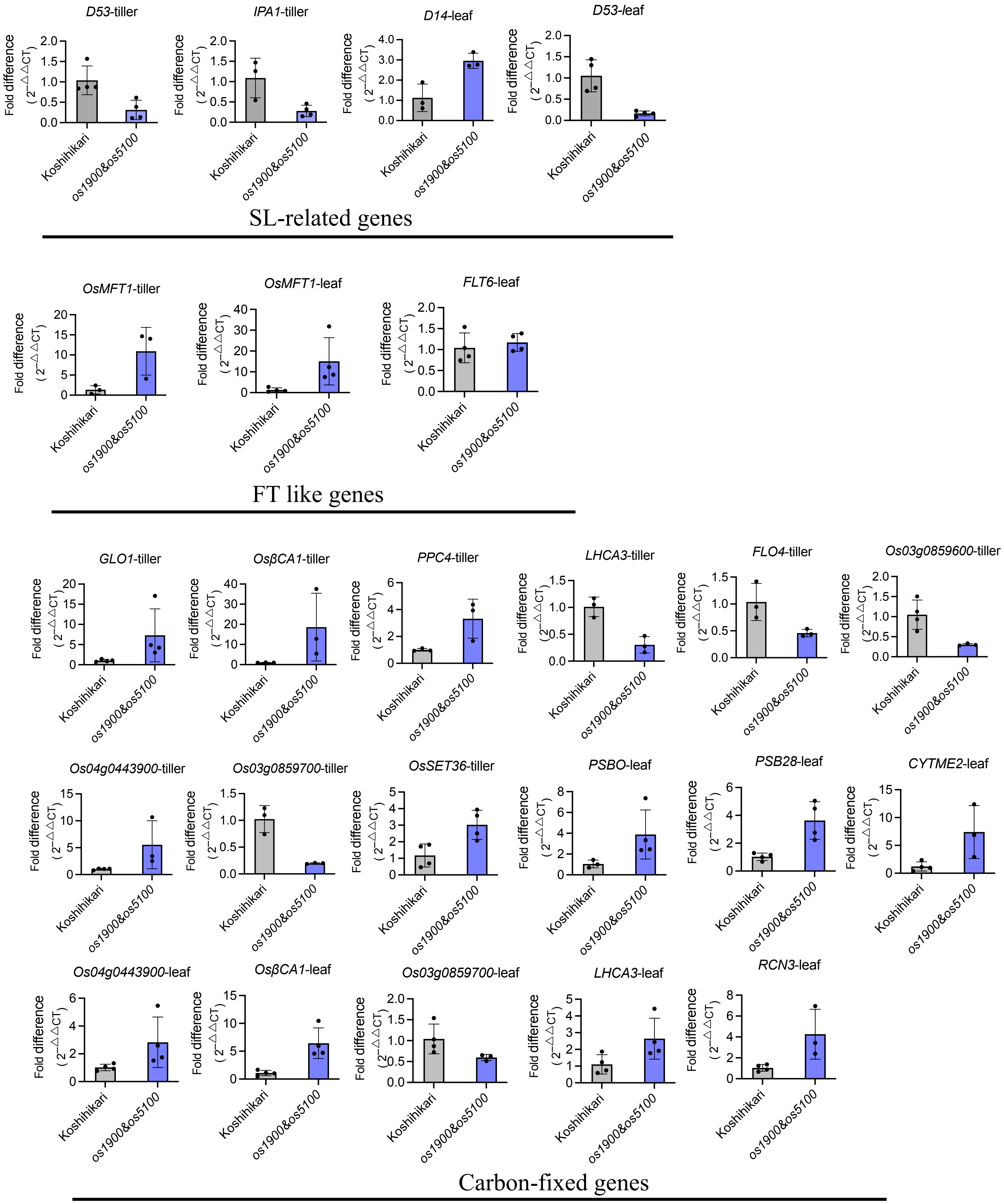

Supplementary Fig. 10 Real time-PCR analysis of selected differentially expressed genes of the first tiller base and leaf in *os1900&os5100* compared with WT, n = 3,4 biologically independent samples, Error bar is SD .

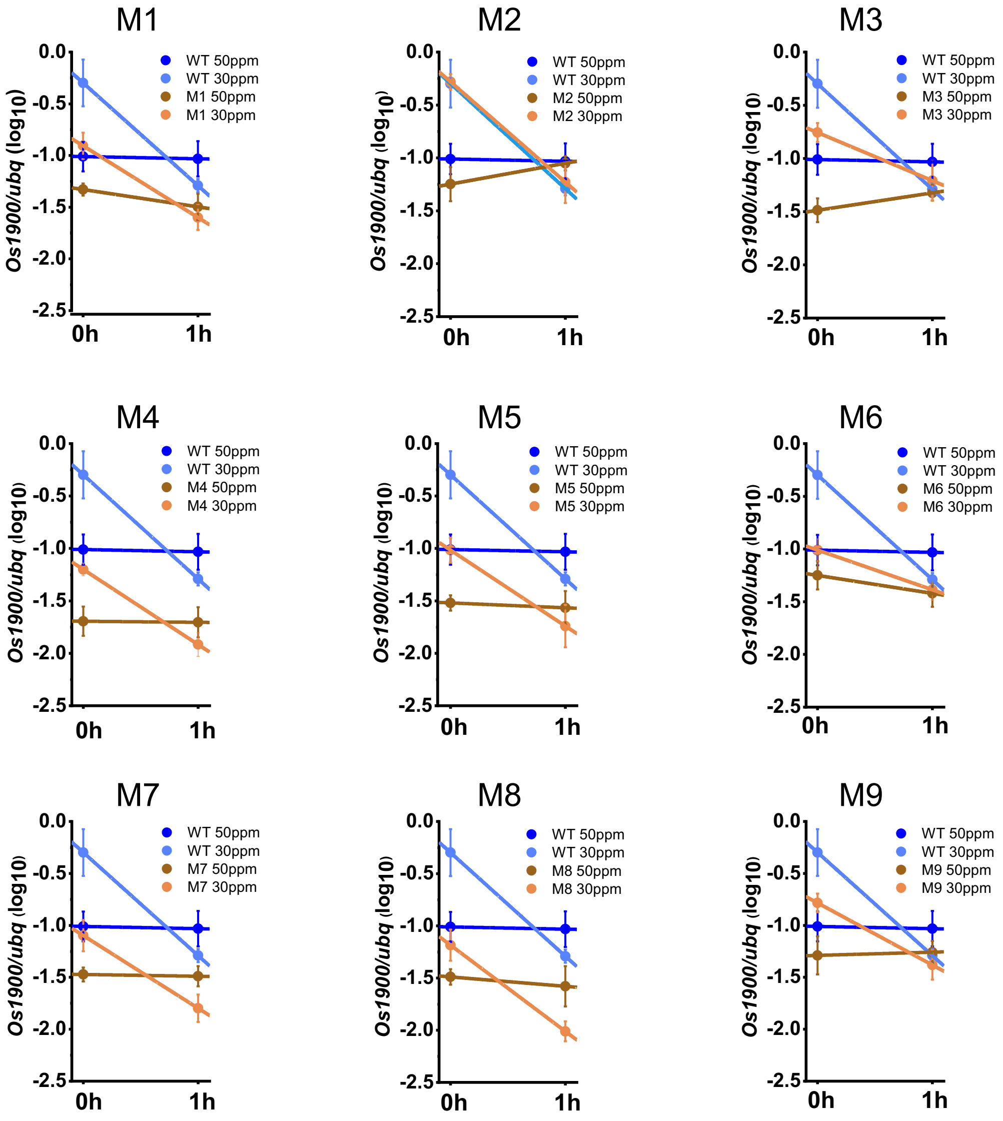

Supplementary Fig. 11 Simple linear models of *Os1900* expression between 0h (50ppm or 30ppm) and 1h (245ppm) in WT and *Os1900* promoter mutants (Slope and intercept, see Supplementary Data 8). Models were made by lm in R. n = 3,4, Error bar is SD.

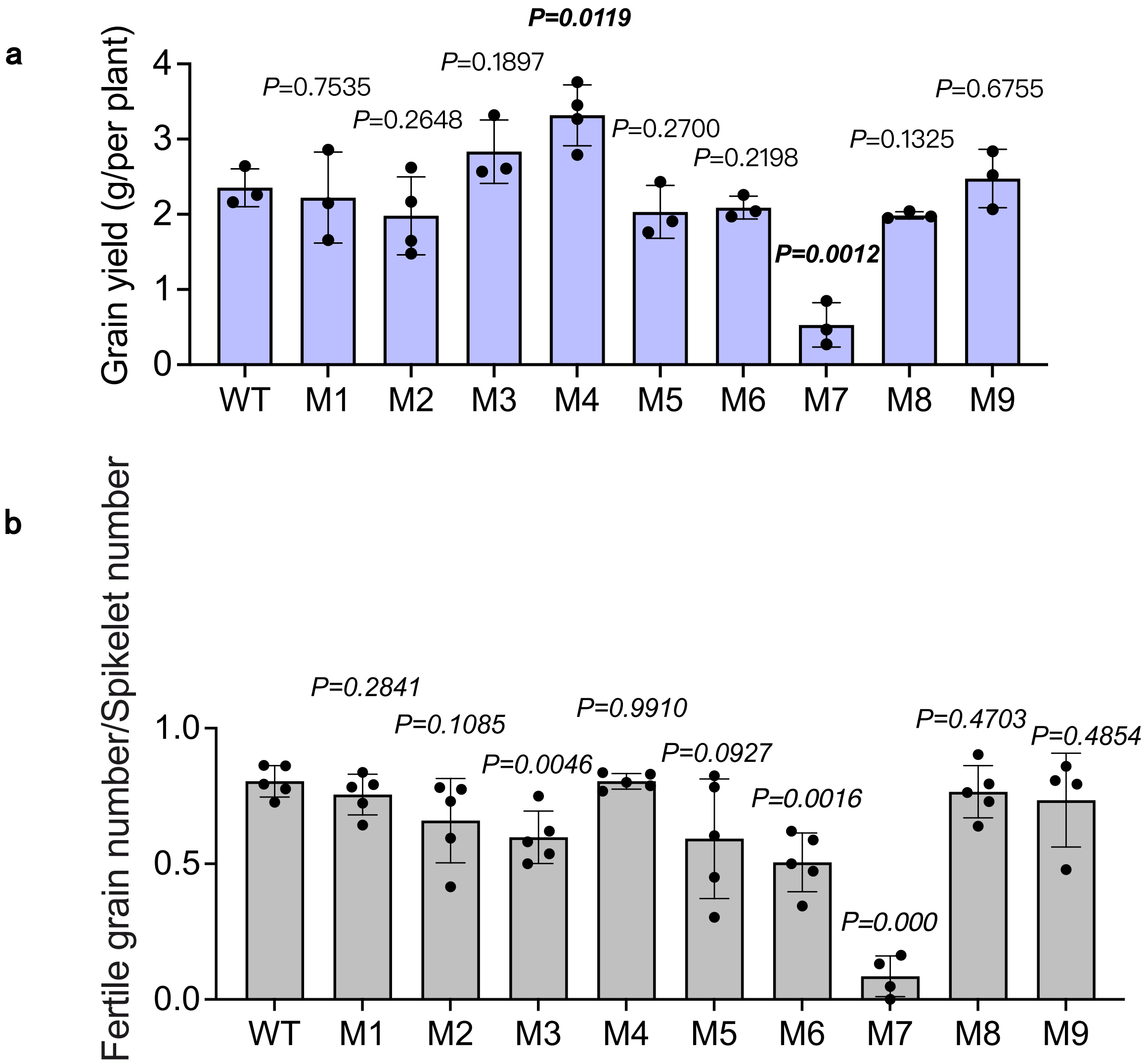

Supplementary Fig. 12 The result of crop yield. Grain yield per plant of *Os1900* promoter mutants (a) and the grain percentage of per panicle (b) under minor fertilization condition. n = 3,4 biologically independent plants (a). n = 4,5 biologically independent plants (b). P values were from Student's t-test (two-tailed) with WT data. Error bar is SD. The spikelets investigated were randomly selected.
