# Supplementary material for: Fertilization controls tiller numbers via transcriptional regulation of a MAX1-like gene in rice cultivation
Source: Nat Commun. 2023 Jun 8;14:3191. doi: 10.1038/s41467-023-38670-8 (PMC10250342; doi:10.1038/s41467-023-38670-8)
Supplement: Supplementary file 3 — Description of Additional Supplementary Files [file 41467_2023_38670_MOESM3_ESM.pdf]

## Description of Additional Supplementary Files:

**Supplementary Data 1:** Experiment combination and sampling time

**Supplementary Data 2:** Raw data_Expression data of 125722 probes in 48 samples.

**Supplementary Data 3:** fdr_sd_radio for 5 combinations test.

**Supplementary Data 4:** Gene list of fdr_0.05 in tiller base and leaf_RNA-seq result

**Supplementary Data 5:** CREs of Os1900 from Plantpan3.0

**Supplementary Data 6:** Common CREs of Os900,Os1400,Os5100

**Supplementary Data 7:** fdr and p.value of Os1900 promoter mutant gene expression under various fertilization condition

**Supplementary Data 8:** Slope and intercept of Os1900 promoter mutant (0h and 1h)

**Supplementary Data 9:** gRNAs and primer sTable S10. Hormone related genes_carbon fixation genes_FT-like genes list for RNA-seqequences

**Supplementary Data 10:** Hormone related genes_carbon fixation genes_FT-like genes list for RNA-seq

**Supplementary Data 11:** LC-MS_MS method
